# From feulgen to modern methods: marking a century of DNA imaging advances

**DOI:** 10.1007/s00418-024-02291-z

**Published:** 2024-05-16

**Authors:** Melike Lakadamyali

**Affiliations:** 1 Department of Physiology, Perelman School of Medicine, University of Pennsylvania, Philadelphia, USA; 2 Department of Cell and Developmental Biology, Perelman School of Medicine, University of Pennsylvania, Philadelphia, USA; 3 Perelman School of Medicine, Epigenetics Institute, University of Pennsylvania, Philadelphia, USA

**Keywords:** Genome organization, Advanced microscopy, Genome labeling

## Abstract

The mystery of how human DNA is compactly packaged into a nucleus—a space a hundred thousand times smaller—while still allowing for the regulation of gene function, has long been one of the greatest enigmas in cell biology. This puzzle is gradually being solved, thanks in part to the advent of new technologies. Among these, innovative genome-labeling techniques combined with high-resolution imaging methods have been pivotal. These methods facilitate the visualization of DNA within intact nuclei and have significantly contributed to our current understanding of genome organization. This review will explore various labeling and imaging approaches that are revolutionizing our understanding of the three-dimensional organization of the genome, shedding light on the relationship between its structure and function.

## Introduction

The discovery of the double helix structure of the DNA by James Watson and Francis Crick in 1953 (Watson and Crick 1953), building on critical X-ray crystallography work by Rosalind Franklin (Franklin and Gosling 1953), was a pivotal moment in molecular biology, as it provided the fundamental understanding of how DNA encodes genetic information and how it is replicated. The discovery of the Feulgen stain came nearly three decades prior to this breakthrough after the pioneering discovery of Robert Feulgen in 1924, a time when the molecular nature of DNA was unknown. The Feulgen stain was a crucial advancement in histology, enabling the visualization of DNA within cells and the observation of nuclear changes during cell division and differentiation (Chieco and Derenzini 1999; Mello and Vidal 2017). This method heralded a new era in the visualization and quantification of DNA.

Our contemporary understanding of human DNA—consisting of 46 chromosomes that all together measure 2 m in length, intricately packed and organized within the cell nucleus as chromatin—has been profoundly shaped by years of innovation in DNA labeling technologies (Lakadamyali and Cosma 2020). These advancements have progressively enhanced our ability to visualize DNA with remarkable specificity and resolution at the cellular level. We now understand that DNA is intricately folded inside the three-dimensional (3D) space of the nucleus to enable communication between distal genomic sequences, including between enhancers and promoters (Misteli 2020). DNA is organized at multiple length scales, beginning with the nucleosome, in which 146 base pairs of DNA wrap around an octamer of histone proteins (Luger et al. 1997). Repeating units of nucleosomes form the beads-on-a-string fiber, also known as the 10 nm fiber (Olins and Olins 1974). The nucleosomes coalesce into irregular domains known as nucleosome clutches at tens of nanometer length scale (Ricci et al. 2015), followed by chromatin nanodomains in the hundreds of nanometer range (Szabo et al. 2020; Nozaki et al. 2017), both of which depend on nucleosome-nucleosome interactions and are disrupted by histone hyperacetylation (Otterstrom et al. 2019; Ricci et al. 2015; Szabo et al. 2020). Additional levels of organization involve topologically associating domains (TADs) (which are made up of multiple chromatin nanodomains) (Nora et al. 2012; Szabo et al. 2020) and A and B compartments (or euchromatin and heterochromatin) (Lieberman-Aiden et al. 2009), as well as chromosome territories (Cremer and Cremer 2010) extending to even larger length scales. This multilevel organization is established and maintained by various mechanisms, including loop extrusion by architectural proteins such as cohesion and CTCF (de Wit and Nora 2023), chromatin anchoring to the nuclear lamina (van Steensel and Belmont 2017), post-translational modifications of nucleosomes that are recognized and bound by various proteins such as the polycomb group proteins (Millán-Zambrano et al. 2022), and phase separation of histones with each other or with other nuclear bodies such as nuclear speckles (Strom and Brangwynne 2019).

This review will focus on the advances in labeling and microscopy methods that have been instrumental in deepening our understanding of the 3D genome organization, charting a journey from the foundational Feulgen stain to the sophisticated imaging techniques of today.

## DNA dyes and light microscopy

**Fig.1:**
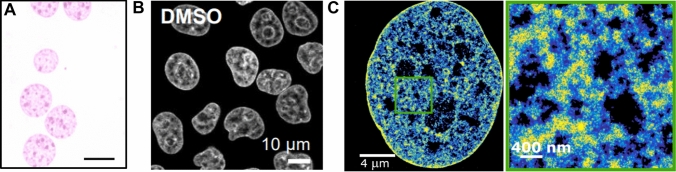
DNA stains and dyes. **A** An example image of the Feulgen stain [adapted from Mello et al. *Acta Histochemica* **119**, 603–609 (2017)] **B** An example confocal image of DAPI stained HeLa cells [adapted from Neguembor et al. *Mol Cell* **81**, P3065–3081.E12 (2021)]. **C** A STORM super-resolution image of DNA labeled using EdC and click chemistry showing nanoscale local variations in DNA density (from low density in blue to high density in yellow; adapted from Otterstrom et al. *Nucleic Acids Res*. **47**, 8470–848 (2019)]

Histological techniques, such as the Feulgen stain (Fig. 1A), have evolved significantly with the advent of fluorescent DNA dyes (Gomes et al. 2018; Suzuki et al. 1997). These dyes emit specific wavelengths of light when excited by a wavelength of light that they absorb, providing exquisite contrast when visualizing DNA. Fluorescent dyes are further attractive as they simplify the visualization of DNA without necessitating complex sample preparation methods, such as transfection with exogenous fluorescent proteins. These fluorescent dyes include those that bind to the DNA major or minor groove such as DAPI (Fig. 1B), Hoechst dyes, SYTO dyes (e.g., various SYTOX variants) and PicoGreen. Additionally, high affinity cyanine dyes that intercalate into DNA such as YOYO-1 and TOTO-1 can be used for staining nucleic acids in fluorescence microscopy. Many of these dyes, including, YOYO-1, SYTO, and PicoGreen have fluorogenic properties in which a large fluorescence enhancement is achieved upon DNA binding, greatly minimizing fluorescence background.

While certain DNA dyes are restricted to fixed cells, many, including Hoechst, SYTO dyes, YOYO, and TOTO series are compatible with living cells (Martin et al. 2005). This enables studying real time DNA dynamics and organization during key cellular processes, such as cell division and differentiation. A recent, notable advancement in this domain is the SiR-DNA dye, which is based on the attachment of carboxylated silicon-rhodamine (SiR) dye to the bisbenzimide core of Hoechst 33,342. This attachment targets the SiR dye to DNA (Lukinavičius et al. 2015). The SiR derivatives are advantageous over other fluorophores as they are excited by far-red light, which induces less phototoxicity in live cell imaging compared with blue or green light illumination. Additionally, the SiR dyes are compatible with super-resolution light microscopy (see below for more details), making the SiR-DNA an attractive DNA dye for live cell and super-resolution imaging although some reports suggest that this stain can induce DNA damage response and impair cell cycle (Sen et al. 2018).

DNA dyes, such as DAPI and Hoechst, have become integral in cellular biology, frequently used as nuclear markers in both live and fixed cell studies. Their applications range from monitoring cell division during developmental stages and analyzing chromosome segregation anomalies to assessing chromatin condensation in varying cell types or during differentiation offering critical insights into cellular processes and genomic organization.

## Fluorescence in situ hybridization, oligopaints, and high throughput genome imaging

**Fig. 2:**
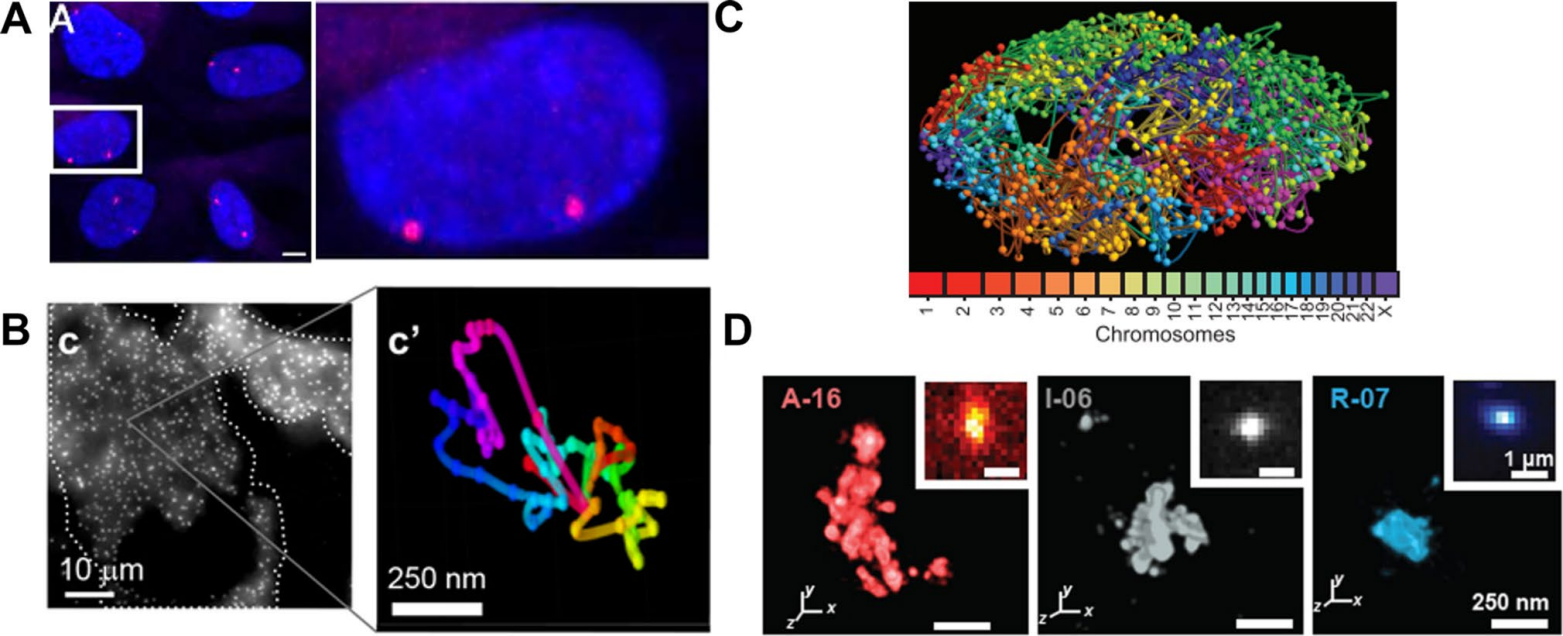
Sequence specific labeling of DNA. **A** Oligopaints image of a 10 kb region at human 4p16.1 [adapted from Beliveau et al. *PNAS ***109**, 21,301–21,306 (2012)]. **B** ORCA reconstruction of the 330 kb BX-C in *Drosophila* embryos [adapted from Mateo et al. *Nature* **568**, 49–54 (2019)]. **C** 3D rendering of chromatin loci color coded by chromosome detected using DNA-MERFISH [adapted from Su et al. *Cell* **182**, 1641–1659 (2020)]. **D** OligoSTORM super-resolution image showing the differences in the level of folding of genomic regions corresponding to different epigenetic states [adapted from Boettiger et al. *Nature* **529**, 418–422 (2016)]

DNA dyes offer many advantages such as ease of use and live cell compatibility. However, these dyes mostly label the entire DNA within the nucleus and lack sequence specificity, which is crucial for understanding the nonrandom organization and folding patterns of chromosomes and genomic domains. DNA fluorescence in situ hybridization or DNA-FISH is a decades old technique (Bauman et al. 1980; Gall and Pardue 1969) that enables visualizing specific genomic sequences and their locations within the nucleus. This technique utilizes fluorescently labeled FISH probes that hybridize with target sequences. Traditionally, these probes have been derived from genomic sequences cloned into vectors such as bacterial artificial chromosome (BAC) plasmids or from custom-synthesized oligonucleotides (oligos) (Finn et al. 2022). Oligo probes offer advantages such as precise targeting and efficient nuclear diffusion due to their shorter length. However, the high costs associated with oligo synthesis limited their use until the development of “oligopaints,” which are more cost-effective at least at small scale (Beliveau et al. 2012) (Fig. 2A). This approach utilizes oligo libraries as a renewable probe source, amplified with fluorophore-conjugated polymerase chain reaction (PCR) primers to create efficient, single-stranded, and strand-specific probes. Oligopaints can visualize genomic regions ranging from a few kilobases (Mateo et al. 2019) to entire chromosomes (Nir et al. 2018; Wang et al. 2016). The use of DNA-FISH and oligopaints have significantly advanced our understanding of the organizational principles of the genome. For instance, they have demonstrated that developmentally important genes are located at the nuclear periphery when silenced and move to the interior when active (Chambeyron and Bickmore 2004). Since DNA-FISH and oligopaints require harsh denaturing treatments, concerns have been raised about the preservation of genomic sequences. This concern is partially mitigated by the bulk level agreement between DNA-FISH and Hi-C (Bintu et al. 2018), the latter of which does not require denaturation. Nonetheless, recent advances that use exonuclease digestion rather than heat denaturation to generate single stranded DNA for efficient probe labeling, including resolution after single-strand exonuclease reaction (RASER)-FISH (Brown et al. 2022), address this potential concern.

Until recently, one major limitation of DNA-FISH and oligopaints was their low throughput. Due to the limited number of spectrally distinct fluorophores and imaging channels, only three to four loci could be visualized in a handful of cells at a time. This limitation has now been overcome with the next generation labeling and imaging methods that drastically improve the throughput of DNA-FISH and oligopaints. Advances in automated image acquisition and automated image analysis have been leveraged to introduce high-throughput FISH (HiFISH) (Finn et al. 2022; Finn and Misteli 2021). HiFISH was used to image up to three loci in an automated and high-throughput manner in hundreds of cells and this imaging could be repeated for hundreds of loci pairs. Application of HiFISH to hundreds of genomic loci revealed a high level of heterogeneity in the relative positioning of these loci within the nucleus (Finn et al. 2019). High throughput genomic imaging technologies has also made it possible to carry out genomewide imaging-based siRNA screens. High-throughput DNA or RNA labeling with optimized oligopaints (HiDRO) is an automated imaging pipeline that enables chromatin interaction measurements in single cells across thousands of samples (Park et al. 2023). Combined with siRNA screens, HiDRO could identify new regulators of genome folding (Park et al. 2023).

The spectral limitation has been overcome by sequential imaging in methods such as Hi-M, where multiple DNA loci are labeled and visualized sequentially in conjunction with RNA transcripts (Cardozo Gizzi et al. 2019). Surprisingly, Hi-M showed that the physical proximity of distal regulatory elements and promoters is not strongly correlated with transcriptional states in a *Drosophila* model system (Espinola et al. 2021). Sequential labeling and imaging also enable tracing the 3D path of genomic DNA, unveiling how different genomic sequences fold in the 3D space of the nucleus. This approach was used to visualize and trace individual TADs within entire chromosomes to show that TADs are organized in two polarized compartments in individual chromosomes (Wang et al. 2016). Optical reconstruction of chromatin architecture (ORCA) is a microscopy approach that uses sequential hybridization and imaging to trace the 3D DNA path with a genomic resolution as fine as 2 kb and a throughput of tens of thousands of cells (Mateo et al. 2019) (Fig. 2B). ORCA is revealing that several loci fold into a complex pattern of stacked, multiloop configurations that bridge enhancers and promoters to regulate transcription (Chen et al. 2023; Hafner et al. 2023).

In sequential imaging methods, such as Hi-M, the number of imaging rounds required scales linearly with the number of loci to be imaged. Clever bar-coding approaches, such as SeqFISH +  (Takei et al. 2021a, b; Takei et al. 2021a, b), DNA-MERFISH (Multiplexed Error Robust Fluorescence in situ Hybridization) (Su et al. 2020)(Fig. 2C), OligoFISSEQ (fluorescence in situ sequencing) (Nguyen et al. 2020), and in situ genome sequencing (IGS) (Payne et al. 2021), have further revolutionized sequential imaging. Most of these methods rely on combinatorial labeling to massively increase the number of genomic loci that can be probed simultaneously in each cell. For MERFISH, for example, the number of loci that can be imaged scales as ~2^N^ where N is the number of hybridization and imaging rounds (Chen et al. 2015). Termed as spatial omics, these techniques can image large-scale genomic landscapes. When combined with RNA-FISH to detect transcripts and immunofluorescence to detect proteins or histone marks, they provide insights into the organizational principles and epigenetic states of genes within the nucleus, offering single-cell and single-allele resolution (Su et al. 2020; Takei et al. 2021a, b; Takei et al. 2021a, b).

## Sequence specific live cell labeling of DNA

DNA-FISH and oligopaints, while invaluable for genomic studies, have a significant drawback: they cannot be used in living cells, thus failing to reveal genome dynamics. Live cell labeling of specific genomic regions is notably challenging (Pradhan et al. 2023). It requires a method that avoids denaturation, and the signal at the target genomic region must be strong enough to be distinguishable from the background. An early solution to these challenges was the use of artificial gene arrays, such as the lac operator (*LacO*)/lac repressor (LacI) system from *Escherichia coli* (Tsukamoto et al. 2000). By inserting arrays of *LacO* into the genome and utilizing a fluorescent protein fusion of LacI, these arrays enable visualization in living cells. However, the *LacO *array’s random insertion offers no control over the insertion site and can also disrupt chromatin organization.

An alternative approach is the ANCHOR system, which involves inserting a *parS* sequence into a specific genomic locus (Germier et al. 2018). The ParB protein, fused to a fluorescent protein, then accumulates on the *parS* sequence due to protein oligomerization. Transcription activator-like effector (TALE) proteins, which can be engineered to bind specific DNA sequences, have also been popular tools for live cell labeling (Miyanari et al. 2013), but it was the advent of clustered regularly interspaced short palindromic repeats (CRISPR)–dCas9 (enzymatically inactive Cas9) that truly revolutionized this field.

CRISPR–CRISPR associated protein 9 (Cas9) has not only revolutionized our ability to precisely edit the genome but the enzymatically inactive Cas9 (dCas9) has quickly been adopted for labeling and visualizing the genome in living cells (Pradhan et al. 2023). Both components of the CRISPR system—the dCas9 fused to a fluorescent protein and the guide RNA —have been used for sequence-specific DNA labeling (Chen et al. 2013; Shao et al. 2016). CRISPR-dCas9 is particularly efficient in labeling the repetitive regions of the genome such as telomeres as only a single guide RNA against the repeating sequence can be used to accumulate and enhance the signal at the genomic locus (Chen et al. 2013). Labeling nonrepetitive sequences poses a greater challenge, initially requiring the delivery of numerous distinct guide RNAs to tile along the locus of interest to sufficiently enhance signal at the target sequence over the background (Chen et al. 2013). However, subsequent advances have started addressing this challenge and improving signal accumulation at genomic sites. Innovations include the use of small multivalent epitope tags, such as SunTag (Neguembor et al. 2018; Tanenbaum et al. 2014), modification of guide RNAs with RNA hairpins (such as MS2 or PP7) (Ma et al. 2015; Qin et al. 2017), and the employment of biomolecular fluorescence complementation with split fluorescent protein systems (Chaudhary et al. 2020; Hong et al. 2018). Additionally, CRISPRainbow, an approach that recruits spectrally distinct fluorescent proteins in a combinatorial fashion by modifying the guide RNA with multiple orthogonal RNA hairpins (MS2, PP7, and boxB) enhanced the ability to simultaneously visualize up to six genomic loci (Ma et al. 2016). Many of the nonrepetitive genome labeling approaches remain challenging to adopt, often requiring complicated strategies that may not always be robust. More developments in easy to implement methods are needed to facilitate the widespread adoption by the scientific community.

Overall, CRISPR-based chromatin labeling stands out as a versatile method. It enables the labeling of multiple genomic regions in different colors and is suitable for various imaging techniques, including live-cell and super-resolution microscopy. This technique has greatly enhanced our ability to visualize and understand the complex organization of the genome in a dynamic cellular environment.

## Super-resolution light microscopy of DNA

The development of fluorescence-based DNA probes and advancements in light microscopy have been instrumental in enhancing our understanding of genome dynamics, organization, and function. Traditional light microscopy, however, faces limitations in resolving the ultrastructure of genes, as most genes occupy spaces inside the nucleus that fall below the spatial resolution limit of conventional light microscopes. While chromatin tracing approaches overcome this limitation by localizing each chromatin segment with nanoscale precision, they tend to provide only a simplified ball-and-sticks representation of chromatin and lack information on the presence of specific factors or epigenetic marks within a gene’s spatial vicinity.

It is, therefore, not surprising that the advent of super-resolution light microscopy has significantly bolstered our understanding of the relationship between chromatin structure and function. The principles of super-resolution microscopy methods, including stimulated emission depletion microscopy (STED), and single molecule-based methods, such as stochastic optical reconstruction microscopy (STORM), and DNA point accumulation in nanoscale topography (DNA-PAINT) have been extensively reviewed elsewhere (Bond et al. 2022; Lelek et al. 2021). Many of these methods have been applied to visualize chromatin both globally and at specific gene loci within the nucleus. These methods often require specialized fluorophores with properties such as high brightness, photostability, and, in some cases, the ability to switch between bright and dark states. While some existing DNA dyes have shown compatibility with super-resolution modalities (e.g., PicoGreen for STORM imaging) (Flors 2013; Flors et al. 2009), their photophysical properties are not ideally suited for such applications, limiting their use to proof-of-concept studies. A significant advancement in this regard is the development of STED-compatible SiR-dye derivatives that can be targeted to various cellular structures including DNA, which has enabled high resolution DNA imaging in both living and fixed cells (Lukinavičius et al. 2015). Another approach involves using nucleotide analogues, such as EdU or EdC, which are incorporated into replicating DNA and, upon fixation, can be tagged with a super-resolution compatible fluorophore through click chemistry (Otterstrom et al. 2019; Xiang et al. 2018) (Fig. 1C). This method also extends to labeling and visualizing RNA distribution in relation to chromatin within cells (Castells-Garcia et al. 2022). Super-resolution microscopy has also been used to visualize nucleosomes by labeling the core histone proteins (e.g. H2B and H3), revealing that nucleosomes assemble into irregular clusters at the nanoscale called nucleosome clutches (~30–100 nm) (Ricci et al. 2015). These structures are cell-type dependent and change in response to chemical as well as mechanical cues (Heo et al. 2023; Otterstrom et al. 2019; Ricci et al. 2015). Moreover, epigenetic marks have been observed to enrich within nanoscale domains and the size of these domains change in diseases such as cancer (Cattoni et al. 2017; Xu et al. 2018, 2020).

Beyond global chromatin visualization, super-resolution microscopy combined with oligopaints in techniques such as OligoSTORM (Fig. 2D) has enabled the visualization of individual genes’ volume and compaction level with nanoscale resolution (Beliveau et al. 2015; Boettiger et al. 2016). Studies using OligoSTORM and other super-resolution approaches have shown that transcriptional inhibition can lead to the compaction of gene structures, especially in highly transcribing genes (Neguembor et al. 2021). Additionally, combining OligoSTORM with chromatin tracing has revealed variability in the borders of topologically associating domains (TADs) at the single-cell level, contrasting with the sharp boundaries observed in sequencing-based approaches, such as Hi-C (Bintu et al. 2018).

Although super-resolution methods such as STED and STORM complement the dynamic view of DNA and chromatin from live cell studies, their ability to visualize multiple targets simultaneously is limited due to the need for specialized fluorophores. While this limitation can be overcome using sequential labeling and visualization using the same fluorophore (Tam et al. 2014; Bintu et al. 2018), this approach is slow and low throughput. An emerging super-resolution approach that overcomes this limitation is expansion microscopy (ExM), in which the sample is physically cross-linked to a swellable hydrogel that allows for isotropic expansion upon absorbing water, leading to a physical magnification of the sample. The principles of ExM have been extensively reviewed elsewhere (Wassie et al. 2019; Wen et al. 2023). ExM, which does not require specialized fluorophores or microscopes, simplifies multicolor imaging and has been increasingly used for chromatin visualization. For example, single-cell evaluation of post-translational epigenetic encoding (SCEPTRE) utilizes ExM to visualize genomic loci via DNA-FISH alongside other targets such as histone marks or RNA PolII (Woodworth et al. 2021). This method has revealed correlations between the abundance of active histone marks and RNA PolII at the single-locus level. Chromatin ExM (or chromExM) employed a 15-fold expansion protocol, revealing how transcription factors such as Nanog and RNA PolII interact with nucleosomes in varying configurations in zebrafish embryos during zygotic genome activation (Pownall et al. 2023).

While ExM holds great potential for multimodal imaging of DNA, RNA, and proteins within the nucleus, caution must be taken to ensure that chromatin is preserved at these nanoscale length scales during the expansion process.

## Electron microscopy of DNA

Electron microscopy (EM) inherently offers much higher spatial resolution than light microscopy. Some of the earliest insights into chromatin organization were gleaned from electron microscopy images of cell nuclei. These images demonstrated that chromatin segregates into two distinct compartments: an electron-dense heterochromatin compartment, typically enriched at the nuclear lamina, and an electron-poor euchromatin compartment. However, the lack of nucleic acid or protein-specific stains compatible with EM initially resulted in low-contrast images that could not reveal the detailed nucleosome-level folding of chromatin.

**Fig. 3:**
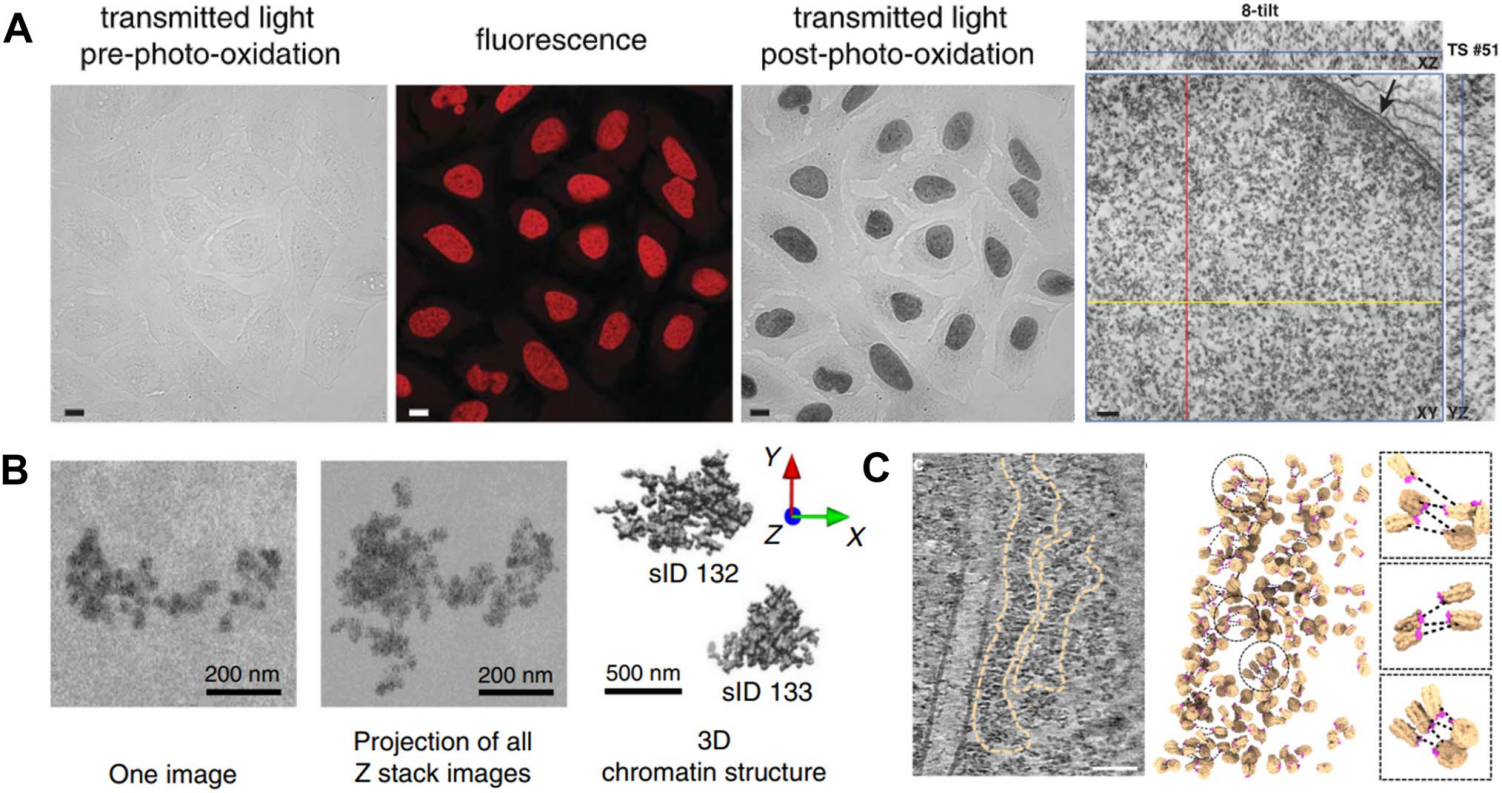
Electron microscopy visualization of DNA and chromatin. **A** U2OS cells stained with DRAQ5, an EM-compatible DNA stain. Photooxidation leads to the appearance of a dark stain under EM [adapted from Ou et al. *Science* **357**, eaag0025 (2017)]. **B** 3D EMISH image of a genomic region showing folding patterns of the chromatin fiber [adapted from Trzaskoma et al. *Nat Commun*. **11**, 2120 (2020)]. **C** A rotated tomographic slice of a CryoET image of native chromatin of T lymphoblast cells and mapping of individual nucleosomes in chromatin fibers [adapted from Hou et al. *Nat Commun* **14**, 6324 (2023)]

A significant advancement in EM of chromatin was the development of an electron microscope-compatible DNA dye (Ou et al. 2017) (Fig. 3A). This dye facilitated the first high-contrast and high-resolution electron microscopy images of chromatin at the nucleosomal level. These ChromEMT (chromatin electron tomography) images corroborated earlier super-resolution findings, showing chromatin as a disordered fiber comprising irregular domains of tightly or loosely packed nucleosomes (Ou et al. 2017). Subsequently, ChromSTEM, which combines DNA-specific staining with scanning electron microscopy, has enabled the analysis of chromatin conformation within chromatin packing domains (Li et al. 2022). It also allows for the quantification of statistical descriptors of chromatin structure relevant to transcriptional activity (Li et al. 2022). Further, serial block-face scanning electron microscopy (SBF-SEM) has recently been combined with in situ hybridization in a method called 3D-EMISH (Trzaskoma et al. 2020) (Fig. 3B). This approach uses biotinylated DNA probes with silver staining to enhance contrast at a specific genomic region for ultrastructural analysis using SBF-SEM (Trzaskoma et al. 2020). This approach has revealed distinct and heterogeneous chromatin-folding structures for megabase scale regions of the genome (Trzaskoma et al. 2020). Some of the drawbacks of these EM-based approaches include the lack of multiple-colors, such as in fluorescence microscopy, and the fact that extensive sample processing and DNA-labeling can potentially compromise sample integrity.

Cryogenic electron microscopy (cryo-EM) methods have also experienced a revolution, bolstered by new detectors and analytical algorithms. More recently, cryo-EM and cryogenic electron tomography (cryoET) has been employed to visualize chromatin in situ at very high resolution across different cell types (Hou et al. 2023; Li et al. 2023; Cai et al. 2018; Jentink et al. 2023; Tan et al. 2023; Beel et al. 2021) (Fig. 3C). Although these cryoET studies are still nascent, they have begun to reveal the heterogeneity in chromatin fiber folding and nucleosome assemblies. They hold significant potential for in situ visualization of native chromatin fibers under various conditions, including both healthy and diseased states.

## Conclusions and outlook

The development of probes for visualizing DNA, including histological stains, fluorescent dyes, and FISH probes, coupled with innovative imaging techniques ranging from confocal to super-resolution microscopy, has challenged traditional textbook models of DNA and chromatin organization within the nucleus. We are currently in an exciting era where multiplexed and multimodal approaches are beginning to offer the first detailed views of thousands of genes in relation to their epigenetic and transcriptional states. These high-throughput methods are poised to unravel the longstanding question of how genome structure relates to gene function during critical processes such as development, reprogramming, and disease onset. However, many of these advanced techniques remain confined to specialist labs, limiting broader accessibility for most biologists and, as a result, potentially slowing scientific progress. The commercialization of “smart” automated and multimodal microscopes that facilitate high-throughput studies of genome organization could significantly accelerate advancements in this field. As we continue to acquire more data, the need for higher resolution, increased sensitivity, and the capability to probe the genome structure–function relationship in living cells will become increasingly crucial, driving the development of new technologies.

In parallel with the advancement of these high-throughput imaging technologies, there is a growing need for sophisticated analytical algorithms capable of extracting meaningful information from the vast amount of imaging data generated. In this context, machine learning, deep neural networks, and artificial intelligence are emerging as powerful tools with immense potential. The application of AI in microscope automation for “smart” data acquisition workflows, as well as in image analysis, will likely yield deeper insights into the interplay between genome organization and gene activity. This integrated approach, combining advanced imaging with AI-driven analysis, is poised to revolutionize our understanding of the fundamental principles governing genome structure, function, and regulation.

## Data Availability

No datasets were generated or analysed during the current study.
